# Conductive Gas Plasma Treatment Augments Tumor Toxicity of Ringer’s Lactate Solutions in a Model of Peritoneal Carcinomatosis

**DOI:** 10.3390/antiox11081439

**Published:** 2022-07-25

**Authors:** Lea Miebach, Eric Freund, Alessandra Lourenço Cecchini, Sander Bekeschus

**Affiliations:** 1 ZIK *plasmatis*, Leibniz Institute for Plasma Science and Technology (INP), Felix-Hausdorff-Str. 2, 17489 Greifswald, Germany; lea.miebach@inp-greifswald.de (L.M.); eric.freund@inp-greifswald.de (E.F.); 2 Department of General, Visceral, Thoracic, and Vascular Surgery, Greifswald University Medical Center, Ferdinand-Sauerbruch-Str., 17475 Greifswald, Germany; 3 Department of General Pathology, State University of Londrina, Rodovia Celso Garcia Cid, Londrina 86051-990, Brazil; alcecchini@uel.br

**Keywords:** cytokines, colorectal cancer, immunogenicity, plasma medicine, reactive oxygen species, ROS

## Abstract

Reactive species generated by medical gas plasma technology can be enriched in liquids for use in oncology targeting disseminated malignancies, such as metastatic colorectal cancer. Notwithstanding, reactive species quantities depend on the treatment mode, and we recently showed gas plasma exposure in conductive modes to be superior for cancer tissue treatment. However, evidence is lacking that such a conductive mode also equips gas plasma-treated liquids to confer augmented intraperitoneal anticancer activity. To this end, employing atmospheric pressure argon plasma jet kINPen-treated Ringer’s lactate (oxRilac) in a CT26-model of colorectal peritoneal carcinomatosis, we tested repeated intraabdominal injection of such remotely or conductively oxidized liquid for antitumor control and immunomodulation. Enhanced reactive species formation in conductive mode correlated with reduced tumor burden in vivo, emphasizing the advantage of conduction over the free mode for plasma-conditioned liquids. Interestingly, the infiltration of lymphocytes into the tumors was equally enhanced by both treatments. However, significantly lower levels of interleukin (IL)4 and IL13 and increased levels of IL2 argue for a shift in intratumoral T-helper cell subpopulations correlating with disease control. In conclusion, our data argue for using conductively over remotely prepared plasma-treated liquids for anticancer treatment.

## 1. Introduction

In the field of applied redox biology, gas plasma-oxidized liquids enable effective ROS/RNS delivery to largely disseminated and/or poorly accessible tumors, including peritoneal carcinomatosis [1,2], intradermal melanoma [3,4,5], and breast cancer [6], successfully reducing tumor burden as already shown in numerous preclinical studies. Carrier liquids are enriched with low-dose oxidants using medical gas plasmas, an emerging technology that drives predominantly short-lived species chemistries to quickly deteriorate and react with long-lived species in the liquid. Applying gas plasmas in oncology is confined to locally restricted, superficially growing, or ulcerating (in palliative settings) tumors [7] and their precancerous stages. In this regard, gas plasma-oxidized liquids are suggested to enlarge the spectrum of oncological patients that could benefit from ROS/RNS-based treatment approaches in the field of plasma onco-therapy. However, the generation of larger volumes using available gas plasma devices is limited to extensive treatment times as of now [8].

Using an argon plasma jet accredited as a medical class IIa device in Europe [9], we recently showed that the formation of ROS/RNS is locally enhanced when the gas plasma jet directly contacts its target [10]. Due to impedance mismatching between the conducting channel and its target, a return stroke with a second ionization front occurs, which serves as an additional ROS/RNS source when operating in conducting mode [11]. Electron attachment reactions [12] and photonic processes due to enhanced VUV emission might contribute to this process [13]. Initially, the clinical implication currently concerns the direct application of medical gas plasmas in acute and chronic wound care and the palliation of cancer patients. However, the conductivity-enhanced deposition of ROS/RNS into liquids eventually translates to the oxidation of liquids in indirect gas plasma treatment regimens, but such superiority has not yet been shown in in vivo models. Hence, in a model of peritoneal carcinomatosis, we aimed to clarify if, at similar exposure times of Ringer’s lactate (Rilac) to gas plasma-derived reactive species (oxRilac), the therapeutic efficacy was enhanced when operating in conducting mode. Tumor weight was assessed after tumor excision, followed by tissue dissociation for analysis of tumor-infiltrating lymphocytes. Moreover, cytokine and chemokine levels were quantified in dissociated tumor samples and the peritoneal lavage of each individual animal to investigate the immunological consequences of the treatment and possible alterations in the tumor microenvironment.

## 2. Materials and Methods

### 2.1. Generation of Gas Plasma-Oxidized Ringer’s Lactate

Sufficient amounts of gas plasma-oxidized Ringer’s lactate (oxRilac) were prepared by treating 20 mL of Ringer’s lactate solutions (Rilac; Braun, Melsungen, Germany) in a 30 mL glass beaker. Argon gas (99.999% purity; Air Liquide, Stralsund, Germany) was excited at the electrode within the kINPen (neoplas, Greifswald, Germany) at 1 MHz at a generated power of 1 W with a flow rate of 3.0 (free mode) or 1.5 (conducting mode) standard liters per minute (slm). The physicochemical properties of the device have been extensively described [9]. Using a computer-controlled *xyz* table (CNC, Iserlohn, Germany), the jet was positioned in contact with the liquid surface (conducting mode) or 1 cm above. The evaporated volume was replaced with equivalent amounts of double-distilled H_2_O to ensure iso-osmolarity. Liquids were stored in aliquots of 1.5 mL at −20 °C for subsequent experiments.

### 2.2. Liquid Analysis

Liquid analysis of oxRilac solutions was performed after one freeze–thaw cycle. Changes in pH were determined using a pH meter (Mettler Toledo, Giessen, Germany). Hydrogen peroxide (H_2_O_2_) was quantified using the Amplex UltraRed Assay (ThermoFisher Scientific, Bremen, Germany) according to the supplier’s instructions. Fluorescence was measured at λ_ex_ 530 nm and λ_em_ 590 nm using a microplate reader (F200; Tecan, Männedorf, Switzerland). Concentrations were calculated against a standard curve.

### 2.3. Cell Culture

CT26 murine colon carcinoma cells (ATCC: CRL-2638; ATCC, Wesel, Germany) were cultured in Roswell Park Memorial Institute (RPMI) 1640 medium (Pan Biotech, Aidenbach, Germany) supplemented with 10% fetal bovine serum, 1% penicillin and streptomycin, and 1% glutamine (all Corning, Kaiserslautern, Germany). Cells were kept under standard culture conditions at 37 °C, 95% humidity, and 5% CO_2_ in a cell culture incubator (CB210; Binder, Tuttlingen, Germany).

### 2.4. Animal Experiments

The animal study protocol was approved by the Institutional Review Board of the State University of Londrina/PR (Brazil) (protocol code 1633.2019.88). Mice were housed under standard laboratory conditions in clean plastic cages with an ad libitum supply of food and water. The 3R principles (reduce, refine, and replace) were applied to minimize animal suffering. A total of 4 × 10^5^ murine colon carcinoma cells (CT26) were injected intraperitoneally to initiate peritoneal carcinomatosis in 8-week-old Balb/C mice. Starting on day 4, mice received intraperitoneal injection of either untreated or gas plasma-oxidized Ringer’s lactate solution (generated in either free or conducting conditions as described above) every second day for a total of five treatment cycles. On day 13, mice were sacrificed, and tumor nodules were excised for downstream analysis.

### 2.5. Tumor Dissociation and Flow Cytometric Analysis

Viable single-cell suspensions of tumors were retrieved using the GentleMacs tumor dissociation kit and the OctaMacs device (both Miltenyi BioTec, Mönchengladbach, Germany) according to the supplier’s instructions. The remaining erythrocytes were lysed using red blood cell lysis buffer (BioLegend, Amsterdam, The Netherlands). After washing, cells were stained with antibodies targeting (conjugate) IA/IE (APC-Fire 750), CD80/86 (APC), CD45 (AF700), F4/80 (PerCp-Cy5.5), CD3 (BV421), CD8 (BV510), CD11c (BV605), CD4 (BV785; all BioLegend, Amsterdam, the Netherlands), MHCI (BUV661; both BD Biosciences, Heidelberg, Germany), and iFluor maleimide 860 (Biomol, Hamburg, Germany) for live–dead discrimination for 30 min at 37 °C. After washing, cells were acquired using flow cytometry (CytoFLEX LX; Beckman-Coulter, Krefeld, Germany) and analyzed using Kaluza 2.1.3 analysis software (Beckman-Coulter, Krefeld, Germany).

### 2.6. Cytokine Analysis

Cytokine levels were analyzed in samples obtained from tumor supernatants after digestion and lavage fluid of each individual animal. Samples were stored at −80 °C for 9 months until cytokine analysis was carried out. Quantification was carried out using a bead-based sandwich multi-analyte assay (BioLegend, Amsterdam, the Netherlands) according to the manufacturer’s instructions. The assay panel contained beads targeted against interferon (IFN) γ, interleukin (IL) 2, IL4, IL5, IL6, IL9, IL10, IL13, IL17A, IL17F, IL22, and tumor necrosis factor (TNF) α. Beads were labeled with fluorescent detection antibodies, and samples were acquired using flow cytometry (CytoFLEX S; Beckman-Coulter, Krefeld, Germany). Subsequent analysis was performed using a specific data analysis software (Vigene Tech, Carlisle, CA, USA). Absolute concentrations of respective analytes were calculated against a standard curve.

### 2.7. Statistical Analysis

Graphing and statistical analysis were performed using Prism 9.4 (GraphPad Software, San Diego, CA, USA). For comparison between groups, *t*-test, one-way ANOVA, or two-way ANOVA was performed as indicated. Data show the mean ± standard error of the mean (SEM) if not indicated otherwise in the figure legends. Levels of significance were indicated as follows: α = 0.05 (*), α = 0.01 (**), and α = 0.001 (***).

## 3. Results

### 3.1. Conducting Gas Plasma Treatment Augments the Delivery of Hydrogen Peroxide into Ringer’s Lactate Solutions

**Figure 1:**
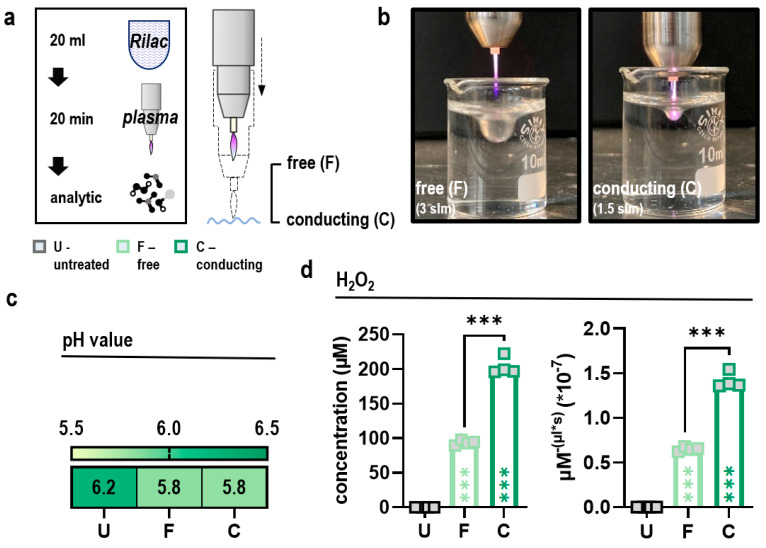
**Conducting gas plasma treatment augments the delivery of hydrogen peroxide into Ringer’s lactate solutions.** (**a**) Schematic overview of gas plasma treatment procedure of Ringer’s lactate (Rilac) in free (F) and conducting mode (C) and subsequent liquid analysis; (**b**) gas plasma treatment in free and conducting mode; (**c**) pH in gas plasma-oxidized Ringer’s lactate (oxRilac); (**d**) absolute concentration of hydrogen peroxide (H_2_O_2_) in oxRilac solutions after treatment in free or conducting mode and delivery thereof in liquids per microliters and seconds. Heat map shows medians. Bar graphs show medians and individual values. Statistical analysis was conducted using one-way analysis of variance (ANOVA) and Tukey’s post hoc testing (*** *p* < 0.001). slm = standard liters per minute; U = untreated; F = free mode; C = conducting mode.

Ringer’s lactate (Rilac) was used as a carrier solution to investigate the impact of conducting gas plasma treatment on the therapeutic efficacy of gas plasma-oxidized liquids. To generate sufficient amounts of oxidized solutions for subsequent experiments, 20 mL of Rilac was exposed to gas plasma for 20 min in free (without contact between jet and liquid surface; F) or conducting (with contact between jet and liquid surface; C) mode. Gas plasma-oxidized Ringer’s lactate (oxRilac) was stored at −20 °C in appropriate volumes. Profiling of species chemistries in the liquid was performed after one freeze–thaw cycle (Figure 1a). Gas plasma treatment was performed using the kINPen, a medical class IIa device, operated with argon at 3 slm (F) or 1.5 (C) slm (Figure 1b). Unsurprisingly in an unbuffered carrier solution, oxRilac solutions experienced a slight decline in pH upon gas plasma treatment (Figure 1c). Medical gas plasmas feature a diverse and highly reactive ROS/RNS chemistry in the gas phase, which is delivered to—but further reacts and deteriorates in—the liquid phase. Gas plasma-oxidized liquids act through rather long-lived chemistries, but attributing selected species to biological effects is under debate. However, hydrogen peroxide (H_2_O_2_) is suggested to play a major role and was found to be increased to a twofold extent in conducting over free mode at equivalent exposure times due to enhanced deposition rates (Figure 1d).

### 3.2. Conducting Gas Plasma Treatment Augments Tumor Toxicity of Ringer’s Lactate Solutions in a Syngeneic Mouse Model of Peritoneal Carcinomatosis in Vivo

**Figure 2:**
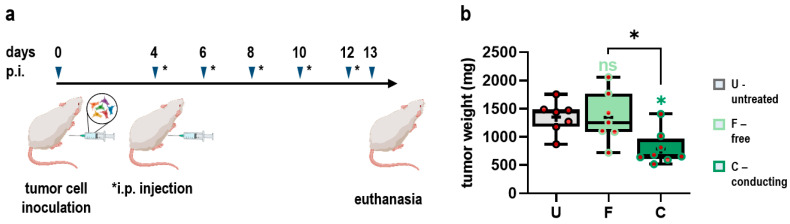
**Conducting gas plasma treatment augments tumor toxicity of Ringer’s lactate solutions in a syngeneic mouse model of peritoneal carcinomatosis in vivo.** (**a**) Experimental procedure; (**b**) 5–95% boxplots showing tumor weight of excised peritoneal tumor nodules. The mean is indicated as +. Statistical analysis was conducted using one-way analysis of variance (ANOVA) and Tukey’s post hoc testing (* *p* < 0.05). ns = nonsignificant; p.i. = post injection; i.p. = intraperitoneal; U = untreated; F = free mode; C = conducting mode.

Next, the therapeutic efficacy of oxRilac solutions exposed to gas plasma in either free or conducting mode was investigated in a syngeneic mouse model of peritoneal carcinomatosis in vivo. Peritoneal carcinomatosis is a severe disease initiated mainly by the metastatic spread of tumors of different primary origins, e.g., gastric, pancreatic, colon, and ovarian cancer, throughout the entire abdominal cavity. Due to the diffuse dissemination of tumor nodules along the peritoneum and abdominal organs, peritoneal carcinomatosis serves as an ideal model for investigating the therapeutic efficacy of gas plasma-oxidized liquids. Moreover, peritoneal lavage using heated (HIPEC) [14] or pressurized chemotherapy (PIPAC) [15] after macroscopic cytoreduction is already approved and applied in oncological treatment regimens, supporting the feasibility of an ROS/RNS-based approach along similar administration routes. In this study, Balb/c mice were engrafted with 4 × 10^5^ CT26 colon carcinoma cells to initiate peritoneal carcinomatosis. Intraperitoneal injection of Rilac, oxRilac (F), or oxRilac (C) was started on day 4 after tumor initiation. Mice received peritoneal lavage every second day in a total of five treatment cycles. Animals were sacrificed on day 13 (Figure 2a). Peritoneal lavage with oxRilac solutions was well tolerated, as adverse side-effects and animal weight loss were not observed (Supplementary Materials Figure S1a). Conducting gas plasma treatment of oxRilac solutions significantly reduced tumor burden of mice suffering from peritoneal cancer, while oxRilac generated in free mode failed to elicit any reduction in tumor growth compared to untreated controls (Figure 2b).

### 3.3. Conducting and Free Gas Plasma-Treated Rilac Equally Enhances Infiltration of Lymphocytes into Tumor Tissues in Vivo

**Figure 3:**
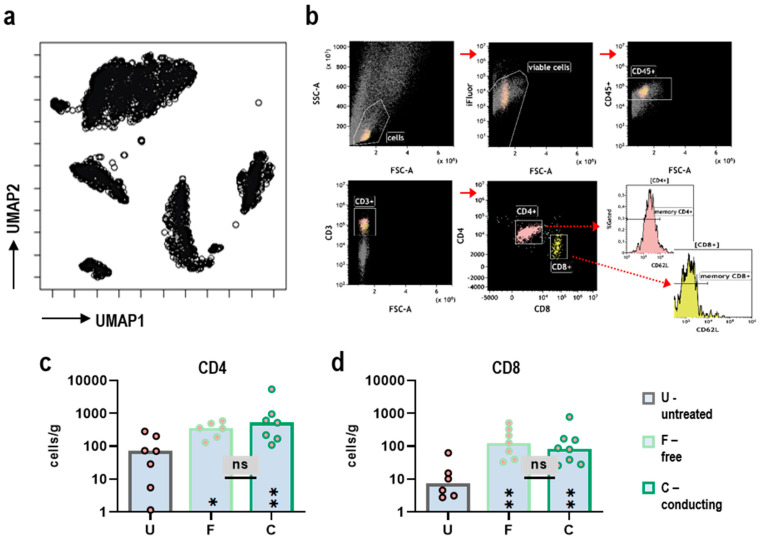
**Conducting and free gas plasma treatment equally enhances the infiltration of lymphocytes into tumor tissues in vivo.** (**a**) UMAP analysis of CD45^+^ leucocytes in dissociated tumor nodules; (**b**) flow cytometry gating strategy to investigate the infiltration of CD4^+^ and CD8^+^ T cells into dissociated tumor nodules; (**c**) the number of CD4^+^ T cells per gram of tumor tissue; (**d**) the number of CD8^+^ T cells per gram of tumor tissue. Bar graphs show medians and individual values. Statistical analysis was conducted using Kruskal–Wallis test and Dunn’s post hoc testing (* *p* < 0.05, ** *p* < 0.01). ns = nonsignificant; U = untreated; F = free mode; C = conducting mode.

The previous findings highlighted the augmented therapeutic efficacy of liquids exposed to gas plasma in conducting mode, giving important implications for such an approach. At equivalent gas plasma treatment times, the conducting mode enabled the delivery of H_2_O_2_ to oxRilac to a twofold greater extent, which correlated with reduced tumor burden in vivo. Complete tumor remission is difficult to achieve with cytotoxic drugs only. Persistent tumor cells might fuel the therapeutic efficacy of oncological regimens, putting the patient at risk for tumor relapse and local or distant metastasis. In this view, a major goal of modern oncological approaches is to draw the immune system’s attention to the tumor side, enhancing the infiltration of immune cells into the tumor microenvironment and enabling a sustained immune response targeted against neoplastic cells. By nature, ROS/RNS-based therapy approaches, such as medical gas plasmas, create an inflammatory-like environment able to trigger antitumor immune responses, e.g., through induction of immunogenic cell death via ROS-induced ER-stress or post-translational modifications of biomolecules creating tumor-specific neoantigens. We hypothesized that the augmented efficacy of conducting treated liquids would also correlate with an enhanced immune cell infiltration into the peritoneal tumor nodules. To this end, excised tumors were dissociated and subjected to flow cytometric analysis of single cells. UMAP algorithm-based analysis calculated from CD45^+^ leucocytes in dissociated tumor tissues indicated the infiltration of different immune cell populations into the tumor (Figure 3a). Surprisingly, quantification of tumor-infiltrating lymphocytes per gram of tumor tissue (Figure 3b) revealed free and conducting treated oxRilac to be equally efficient in attracting CD4^+^ (Figure 3c) and CD8^+^ T cells (Figure 3d) to the tumor side with significant differences compared to untreated controls.

### 3.4. Cytokine Profiles in the Tumor Microenvironment Are Altered after oxRilac Peritoneal Lavage

**Figure 4:**
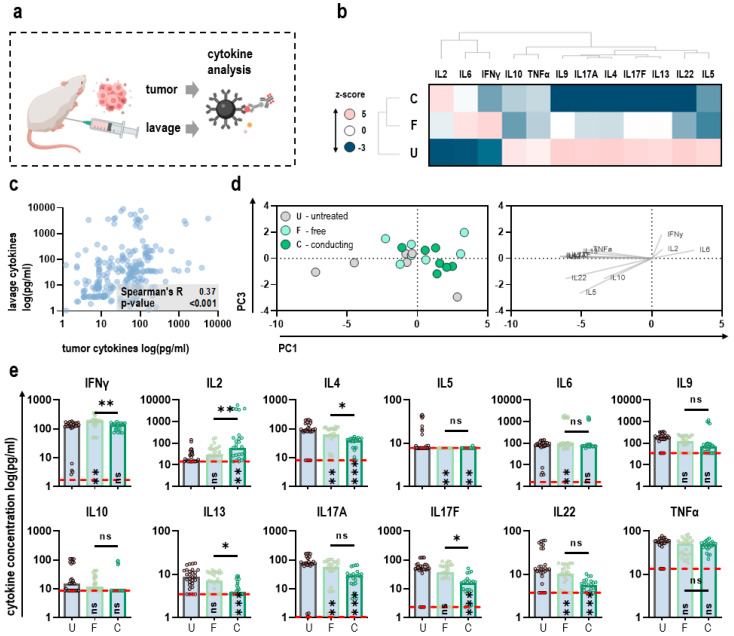
**Cytokine profiles in the tumor microenvironment of mice receiving intraperitoneal injections of free or conducting oxRilac.** (**a**) Schematic overview of samples obtained for cytokine analysis; (**b**) WPGMA-weighted hierarchical clustering calculated from z-scored intratumoral cytokine concentrations; (**c**) Spearman’s correlation between intra- and extratumoral (lavage) cytokine concentrations; (**d**) principal component analysis (PCA) calculated from z-scored intratumoral cytokine concentrations of individual animals showing PC scores and loadings; (**e**) absolute intratumoral cytokine concentrations. Bar graphs show medians and individual values. The limit of detection is indicated as a dashed red line. Statistical analysis was conducted using one-way analysis of variance (ANOVA) and Tukey’s post hoc testing (* *p* < 0.05, ** *p* < 0.01, *** *p* < 0.001). ns = nonsignificant; U = untreated; F = free mode; C = conducting mode.

We aimed to further investigate the immunological consequences of oxRilac peritoneal lavage in the tumor microenvironment by mapping intratumoral cytokine profiles after tumor dissociation and likewise in ascites (lavage) of each individual animal using a bead-based multianalyte assay (Figure 4a). WPGMA-weighted hierarchical clustering of z-scored cytokine concentrations across all groups underlined the immunogenic consequences of oxRilac peritoneal lavage with slight differences between free and conducting mode (Figure 4b), along with similar tendencies in tumor and lavage samples (Figure 4c). Principal component analysis (PCA) calculated from z-scored cytokine concentrations for each individual animal highlighted differences in intratumoral levels of interleukin (IL)2 and interferon (IFN)γ, as well as IL6 between oxRilac receiving mice and untreated controls (Figure 4d). Interestingly, T cell- and NK-cell activating IL2 was exclusively increased in the conducting group, while animals in the free group showed a significant increase in levels of IFNγ and IL6. A significant reduction was found for T_H_2- and T_H_17-related cytokines for both free and conducting treated oxRilac. Concentrations of cytokines related to T_H_9 responses, such as IL9 and IL10, remained largely unchanged (Figure 4e).

## 4. Discussion

ROS/RNS exhibit pleiotropic roles in physiological signaling pathways but damage cells when applied at supraphysiological levels. Their dual role is outlined by the concept of hormesis and exploited by ROS/RNS-based therapy approaches in clinical oncology, including radiotherapy or photodynamic therapy (PDT) [16]. In 2013, medical gas plasma technology, a multicomponent tool driving mainly short-lived ROS/RNS chemistries, was initially accredited as a novel physics-based therapy for treating chronic wounds [17], but head and neck cancer patients already benefited from such an approach [18]. With available gas plasma devices, however, the repeated or frequent therapeutic application of gas plasmas in direct regimens is largely limited to superficially growing, locally restricted cancers. The obligate need for repetitive treatment cycles over a long time period [7] would imply repeated surgery in the case of internal neoplasms, which is ethically not justifiable. Minimally invasive, endoscopic applications could be promising [19], but more comprehensive research is needed on how ROS/RNS chemistries of such devices are influenced under ambient conditions present, e.g., in the gastrointestinal tract, or by CO_2_ insufflation required for such an approach.

Interestingly, in the same year of accreditation, it was first reported for an argon plasma jet that oxidized cell culture medium successfully reduced the tumor burden of chemoresistant ovarian cancer in a non-orthotopic model in vivo [20]. The findings of Utsumi and colleagues opened the door to investigate novel, minimally invasive administration routes to deliver ROS/RNS to the tumor side of internal, disseminated neoplasms, which was recently extended by the therapeutic application of oxidant-enriched hydrogels [21]. As a current major limitation, oxidant enrichment of carrier liquids requires extensive treatment times, and several attempts have been made to improve the delivery of ROS/RNS into the liquid in eligible reactors [8]. For example, approaches that increase the gas plasma–liquid surface area based on liquid bubbling [22] or microdroplets featuring a high surface-to-volume ratio [23] are promising. In a bedside-to-bench study, we could recently show that species formation of an argon plasma jet is locally enhanced if the visible gas plasma jet plume is operated in direct contact with its target (conducting mode) [10]. The question was whether this finding ultimately translates to the administration of gas plasma-oxidized liquids and, hence, if, at similar exposure times of a carrier liquid to gas plasma, its therapeutic efficacy could be increased via enhanced ROS/RNS deposition rates. To this end, we investigated the therapeutic efficacy of medical-grade Ringer’s lactate, oxidized in free (without contact between jet and target; F) and conducting mode (with contact between jet and target; C) in a syngeneic mouse model of peritoneal carcinomatosis. In view of future clinical implementation, the chosen carrier solution is critically important. Considering translational research from a clinician’s perspective, regulatory issues, and the fact that biological ingredients supplementing cell culture media have marked scavenging effects, which even dampen the efficacy of the treatment [24], the latter is clearly not eligible for such an approach.

Conductivity enhanced deposition of H_2_O_2_ twofold compared to the free mode. In gas plasma-oxidized liquids intended to be used in oncological settings, H_2_O_2_ is suggested to play a central role in mediating biological effects [25,26]. Synergistic effects with nitrite have been discussed, as the reaction between both can form highly reactive peroxynitrites [27]. A broad range of tumor entities have been successfully targeted using gas plasma-oxidized liquids in vitro and in vivo, including lung cancer [28], melanoma [29], pancreatic cancer [30], osteosarcoma [24], glioblastoma [31], and breast cancer [32]. In many of those studies, apoptosis [33] was found to be the central cell death mechanism, but recent reports also suggested that other cell death pathways, including necroptosis [34] and autophagy [35], play a role. As shown in our study, the therapeutic efficacy of gas plasma-oxidized liquids can be significantly enhanced when operating in conducting mode. Due to enhanced H_2_O_2_ deposition rates, the latter significantly reduced tumor burden in a model of peritoneal carcinomatosis, while peritoneal lavage with Rilac oxidized in free mode did not. Providing similar treatment conditions, e.g., liquid volume and beaker shape, an almost linear increase in H_2_O_2_ deposition can be assumed for regular kINPen jet treatments [36]. The clear advantage of conducting gas plasma treatments for indirect gas plasma application is that treatment times could be cut in half compared to the free mode while maintaining similar therapeutic efficacy. Accordingly, the applicability of such an approach is greatly facilitated. In addition to H_2_O_2_-mediated effects, Tanaka and colleagues could recently show that the lactate chemistry in plasma-oxidized Ringer’s lactate solutions contributes to the observed antitumor effects [37]. Differences in free and conducting plasma treatment were not investigated in this regard, but might further enhance the therapeutic efficacy of conductively treated liquids.

The majority of conventional chemotherapeutics applied in clinical oncology elicit their cytotoxicity by interfering with a broad range of proteins that affect DNA synthesis and replication. However, complete tumor remission is difficult to achieve in such settings, putting patients at risk of being left with minimal residual disease, causing tumor relapse and treatment failure. To this end, enhancing the immune system in oncological treatment regimens has been a major breakthrough. In addition to an array of biologicals and checkpoint inhibitors paving their way into the clinic, a screening of 22 alkylating agents revealed some to have marked immunogenic effects [38], which led to the concept of immunogenic cell death (ICD). Cells undergoing ICD release and express damage-associated molecular patterns (DAMPs), e.g., calreticulin, heat-shock protein 70 and 90, ATP, and/or HMGB1, which attract antigen-presenting cells (APCs) to the tumor side [39]. Activated APCs can present processed tumor material to T cells in draining lymph nodes, which might subsequently propagate an effector T-cell response against cancer-specific antigens. ROS/RNS-based approaches such as radiotherapy and photodynamic therapy, as well as medical gas plasmas, have been shown to act as bona fide ICD inducers [40,41,42,43], which is further supported by the creation of neoantigens via oxidation of biomolecules [44]. Along similar lines, gas plasma-oxidized liquids have been shown to increase CRT and HMGB1 expression, as well as ATP release on and by colorectal cancer cells [45] and others, followed by increased phagocytosis and expression of maturation markers on monocyte-derived dendritic cells [46,47]. Surprisingly, infiltration of CD4^+^ and CD8^+^ T cells did not differ between free and conducting regimens in our study, indicating lower amounts of H_2_O_2_ to be sufficient to increase tumor cell immunogenicity while not being able to control and reduce tumor growth overall. Slight differences could, however, be observed after mapping intratumoral cytokine profiles quantified for individual mice.

In the conducting group, a marked increase in lymphocyte-activating IL2 was found. The ability of IL2 to expand the patient’s NK cell compartment, induce T-helper cell function, and boost the reactivity of previously generated cytotoxic T lymphocytes even gave rise to the idea of IL2-based immunotherapy years before checkpoint inhibitors were implemented in the clinic [48,49,50]. With other limitations, recombinant IL2, called aldesleukin, was FDA approved for treatment of metastatic renal cancer in 1992 and metastatic melanoma in 1998, underlining the importance of IL2 in generating an antitumor immune response. By contrast, the free group differed mainly in intratumoral levels of IFNγ and IL6. Functional consequences of IFNγ signaling are complex, with both positive and negative regulatory activities reported. The presence of IFNγ in the TME has been linked to upregulation of MHC I and antigen-processing molecules, with antiproliferative and antiangiogenetic effects accompanied by effector T-cell recruitment to the tumor side [51,52,53]. On the other hand, IFNγ has also been shown to induce upregulation of immune-suppressive molecules, including PDL1 and indolamine-2,3-dioxygenase (IDO), as well as promote T cell apoptosis in vivo [54,55]. Likewise, IL6 is found in diverse TMEs and has been linked to tumor development and therapy resistance in various cancer entities via stimulating immune suppressive signals [56,57]. In both free and conducting groups, a significant reduction was found for T_H_2- and T_H_17-related cytokines, while T_H_9- related cytokines, such as IL9 and IL10, remained largely unchanged. As a limitation, we did not correct for different tumor volumes so that relative intratumoral concentrations might be increased, especially in the conducting group. Overall, increased levels of IL2 in the TME of the conducting group might be associated with enhanced antitumor immunity, supporting the cytotoxicity of this regimen, but this remains to be elucidated.

In addition to aiming for maximal therapeutic efficacy, oncological treatments have to be safe. The present study focused on tumor-specific and tumor-immunological consequences of the treatment. Effects on nonmalignant cells constituting the peritoneal TME, e.g., mesothelial cells lining the peritoneal cavity or stromal fibroblasts, were not investigated. However, peritoneal lavage using oxRilac solutions did not induce animal weight loss or adverse side-effects, emphasizing the good tolerability of such an approach, as already supported by many studies [6,30,58]. Notwithstanding, nonmalignant cells present in the TME are often not innocent bystanders but can be hijacked by tumor cells to aid in tumor progression, invasion, and metastasis. In addition to immune cell suppression, desmoplastic reactions of tumor resident stromal fibroblasts can occur, e.g., pancreatic stellate cells (PSC) in the context of pancreatic ductal carcinoma, which are a major cause of therapy resistance and poor patient outcome. Direct and indirect plasma treatment regimens have recently been shown to counteract PSC activation and extracellular matrix remodeling [46,59,60,61], as well as exuberant proliferation and activity of stromal fibroblasts after peritoneal surgery and prolonged inflammation [62], which translates to conditions in the TME that might be additionally beneficial after cytoreductive surgery in PC.

The present study translates previous knowledge on the efficacy of conducting gas plasma treatments to the generation and application of gas plasma-oxidized liquids. Conductivity enhanced the therapeutic efficacy of oxRilac solutions in a model of peritoneal carcinomatosis, giving important implications for indirect gas plasma applications in the future. Notwithstanding, from a translational perspective, the opportunity to mimic plasma-oxidized liquids with chemically manufactured solutions would greatly facilitate their routine application as an oncological strategy.

## 5. Conclusions

The present study emphasizes the advantage of gas plasma treatment in conducting mode and translates previous knowledge concerning the augmented therapeutic efficacy to the indirect administration of gas plasma-derived ROS/RNS through gas plasma-oxidized liquids. At similar gas plasma exposure times of Ringer’s lactate, the delivery of tumor-targeting H_2_O_2_ could be increased twofold, correlating with a significant reduction in tumor burden in mice suffering from peritoneal carcinomatosis. Peritoneal lavage with oxidized liquids moreover increased lymphocyte infiltration into the tumor nodules, accompanied by considerable increases in intratumoral IL2 in the conducting group.

## Data Availability

The underlying data of this study are available from the corresponding author upon reasonable request.

## Supplementary Materials

The following supporting information can be downloaded at: https://www.mdpi.com/article/10.3390/antiox11081439/s1, Figure S1: Peritoneal lavage using oxRilac solutions did not induce animal weight loss.
