# Supplementary material for: Conductive Gas Plasma Treatment Augments Tumor Toxicity of Ringer’s Lactate Solutions in a Model of Peritoneal Carcinomatosis
Source: Antioxidants (Basel). 2022 Jul 25;11(8):1439. doi: 10.3390/antiox11081439 (PMC9331608; doi:10.3390/antiox11081439)
Supplement: Supplementary file 1 [file antioxidants-11-01439-s001.zip › antioxidants-1836252-supplementary.pdf]

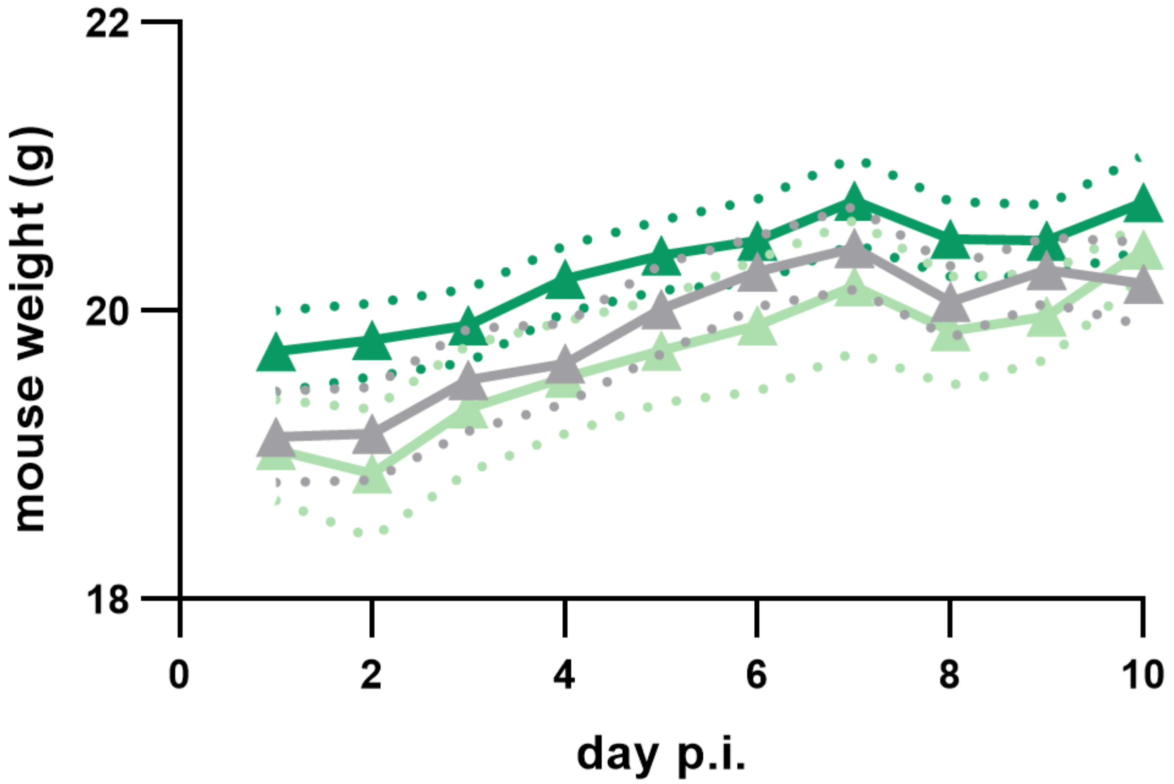

Figure S1. Peritoneal lavage using oxRilac solutions did not induce animal weight loss. animal weight of the different groups (grey: untreated Rilac; light green: gas plasma-treated Rilac; dark green: conductively gas plasma-treated Rilac) monitored throughout the experimental procedure. Graph shows mean ± standard error of the mean.

Figures

Figure 1
